# Using a brief web-based 5A intervention to improve weight management in primary care: results of a cluster-randomized controlled trial

**DOI:** 10.1186/s12875-021-01404-0

**Published:** 2021-04-02

**Authors:** Franziska D. Welzel, Jonathan Bär, Janine Stein, Margrit Löbner, Alexander Pabst, Melanie Luppa, Thomas Grochtdreis, Anette Kersting, Matthias Blüher, Claudia Luck-Sikorski, Hans-Helmut König, Steffi G. Riedel-Heller

**Affiliations:** 1 Institute of Social Medicine, Occupational Health and Public Health (ISAP), Medical Faculty, University of Leipzig, Philipp-Rosenthal-Straße 55, 04103 Leipzig, Germany; 2 Integrated Research and Treatment Centre (IFB) AdiposityDiseases, Leipzig University Medical Center, Leipzig, Germany; 3 Department of Health Economics and Health Services Research, University Medical Centre Hamburg-Eppendorf, Hamburg, Germany; 4 Clinic for Psychosomatic Medicine and Psychotherapy, University of Leipzig, Leipzig, Germany; 5 Department of Endocrinology, Nephrology, Rheumatology, University Hospital Leipzig, Leipzig, Germany; 6 SRH University of Applied Sciences Gera, Gera, Germany

**Keywords:** Obesity, 5As counseling, Primary care, Provider-patient-interaction, CRCT

## Abstract

**Background:**

The primary health care setting is considered a major starting point in successful obesity management. However, research indicates insufficient quality of weight counseling in primary care. Aim of the present study was to implement and evaluate a 5A online tutorial aimed at improving weight management and provider-patient-interaction in primary health care. The online tutorial is a stand-alone low-threshold minimal e-health intervention for general practitioners based on the 5As guidance for obesity management by the Canadian Obesity Network.

**Methods:**

In a cluster-randomized controlled trial, 50 primary care practices included 160 patients aged 18 to 60 years with obesity (BMI ≥ 30). The intervention practices had continuous access to the 5A online tutorial for the general practitioner. Patients of control practices were treated as usual. Primary outcome was the patients’ perspective of the doctor-patient-interaction regarding obesity management, assessed with the Patient Assessment of Chronic Illness Care before and after (6/12 months) the training. Treatment effects over time (intention-to-treat) were evaluated using mixed-effects linear regression models.

**Results:**

More than half of the physicians (57%) wished for more training offers on obesity counseling. The 5A online tutorial was completed by 76% of the physicians in the intervention practices. Results of the mixed-effects regression analysis showed no treatment effect at 6 months and 12 months’ follow-up for the PACIC 5A sum score. Patients with obesity in the intervention group scored lower on self-stigma and readiness for weight management compared to participants in the control group at 6 months’ follow-up. However, there were no significant group differences for weight, quality of life, readiness to engage in weight management, self-stigma and depression at 12 months’ follow-up.

**Conclusion:**

To our knowledge, the present study provides the first long-term results for a 5A-based intervention in the context of the German primary care setting. The results suggest that a stand-alone low-threshold minimal e-health intervention for general practitioners does not improve weight management in the long term. To improve weight management in primary care, more comprehensive strategies are needed. However, due to recruitment difficulties the final sample was smaller than intended. This may have contributed to the null results.

**Trial registration:**

The study has been registered at the German Clinical Trials Register (Identifier: DRKS00009241, Registered 3 February 2016).

## Background

Obesity represents a global and increasing challenge for healthcare providers. According to the World Health Organization (WHO), the worldwide prevalence of obesity nearly tripled between 1975 and 2016, with an estimated number of over 650 million (13%) adults with obesity in 2016 [1]. In Germany, almost one in four adults (23% of men and 24% of women) can be classified as obese [2]. This increase is alarming considering the wide range of health-related risks that are associated with obesity [3, 4]. Obesity is associated with a lower life expectancy and a risk factor for several somatic diseases such as hypertension, coronary heart disease and diabetes [5] as well as a range of mental disorders [6], with individuals with obesity frequently experiencing discrimination and stigmatization [7]. Worldwide healthcare costs for patients with obesity were found to be approximately 30% higher than for individuals without obesity, with direct obesity costs resulting in around 0.7% to 2.8% of a country’s total healthcare expenditures [8]. Consequently, tackling obesity is a major concern of the public health sector, characterized by a growing need for effective evidence-based interventions for obesity treatment and prevention.

Concerning the management of obesity and related medical conditions, the primary care setting plays a crucial role in the delivery of care. As gatekeepers to the healthcare system, general practitioners (GPs) are among the first to be contacted when secondary diseases and comorbidities arise. Considering that their interactions with patients take place on a regular basis, GPs may be specifically suited to address overweight and obesity. An important factor for patients with obesity to attempt weight loss may thereby lie in their health care professionals’ communication of overweight status, as well as their advice to lose weight [9, 10]. However, hindering aspects of obesity management in the primary care setting have been identified considering both GPs and patients [11–15]. Barriers include GPs’ lacking recognition of obesity [12] and insufficient amounts (42%) of GPs perceiving themselves to be well prepared for treating patients with excess body weight [13]. On the other hand, research indicates that patients are unlikely to address weight when consulting a GP due to tight time constraints or seem to prefer other health care professionals, such as personal trainers or dieticians above medical practitioners for weight management [14]. Another important aspect in this regard is the physician–patient-relationship. Physician–patient-interactions in primary care have previously been linked to patient’s experience of chronic care [16] and the self-management behavior of patients such as uptake of preventive activities, management of personal distress and medication adherence [17–20]. In turn, higher patient motivation and self-management behavior have been associated with better self-reported and physical health outcomes [20, 21]. Therefore, effective tools are needed to improve communication and management of obesity in primary care. Recently, the 5A model has been proposed as a framework to improve weight counseling in the primary care context [22]. The 5A was initially developed as a tool for counseling on smoking cessation and has later been adapted for the context of weight management counseling [23]. The 5As comprise the assessment of risk, asking about readiness to lose weight, advising on change, assisting in establishing interventions, and arranging for follow-up visits. Previous research has promoted the 5As as an effective tool to increase quit rates and to enhance motivation in smoking cessation [24–26]. In the context of counseling on weight GPs seem to be inconsistent in using the 5As with their patients [27]. While GPs routinely ask and advise patients about losing weight, they rarely engage in the strategies of assess, assist and arrange [27].

The Canadian Obesity Network (CON) has proposed a standardized framework for a 5A-based weight management counseling, the “5As of Obesity Management” [28]. Comprising handout material and an online tutorial, this framework synthesizes the Canadian Obesity Guidelines [29] with the concept of the 5As [28]. It provides an elaborated and clear guidance in weight management counseling and motivates physicians to focus primarily on improvements in health with their patients [22, 28]. While weight loss is an important goal to improve health and well-being in many patients, it may not be equally suitable for all patients with overweight or obesity as the amount of excess body weight alone may not be a reliable indicator of poor health per se [9, 22, 30].

Previous research reporting positive effects of 5A-based interventions has mostly assessed effects in short-or medium-term [31–33], whereas research on long-term effects of 5A-based counseling has been more inconclusive [34, 35]. While one study found no change in the overall number of counseling dialogues regarding physical activity 6 months after a 5A training [34], another study reported a significant change in patients’ weight 12 months post-intervention [35]. To our knowledge, there are no results on the long-term effectiveness of the 5As of weight counseling for the German primary care context.

As competing demands on time hinder physicians to integrate preventive and counseling services in their day-to-day work [36, 37] a brief web-based 5A application that allows for a flexible and customized use may be specifically suitable in order to improve GPs counseling technique. A web-based tutorial with brief educational information and a clear design has the advantage of providing GPs with relevant information on weight counseling right at the time they are needed (e.g. in preparation of preventive services or in between patients), which in turn may help GPs to integrate such weight counseling services to a greater extent.

The objective of this study was to investigate the effectiveness of a short and flexibly usable 5A-based online tutorial for GPs. The 5A-based online intervention aims at improving the quality of care and the physician–patient-interaction in the context of weight management. Consequently, the effectiveness of the 5A-based intervention was assessed via the patients’ perception of the quality of care provided to them.

## Methods

### Setting and trial design

The INTERACT study comprised a cluster-randomized controlled trial (cRCT) with an intervention condition (IG) and a waiting list condition (CG). The trial was set within the primary care setting in the region of central Germany. GPs from registered practices were invited to participate in the INTERACT study and to recruit eligible patients. Randomization took place at the level of GPs, which served as the clustering variable. GPs allocated to the IG received access to a 5A online tutorial which offers education on weight counseling according to the “5As of Obesity Management” by the Canadian Obesity Network [28]. GPs allocated to the CG followed the care-as-usual protocol, receiving access to the 5A intervention only after the trial was completed.

### Ethical considerations

The ethics committee of the University of Leipzig (reference number 248/15-ff) approved the study. Written informed consent was obtained from all GPs and participating patients. The INTERACT study was conducted according to the latest version of the Declaration of Helsinki and the Guidelines for Good Clinical Practice [38] as well as international and local laws.

### Recruitment

GPs were recruited between January and May 2016 based on a primary care physician network, as previously established by the Institute of Social Medicine, Occupational Health and Public Health of the University of Leipzig (ISAP). GPs from the IG received continuous access to the 5A online tutorial. They were asked to complete the tutorial within two months after receiving login data. GPs from the CG had no access to the online tutorial throughout the trial and received login data to the 5A tutorial 6 months after the trial ended. GPs were granted CME points (continuing medical education) upon successful completion of the tutorial. Participating GPs received a fixed allowance of 80 Euros per recruited patient. GPs of both groups were asked to fill out questionnaires following recruitment (baseline, BL), as well as at 12-month follow-up.

The patient sample included adult patients with obesity recruited through participating GPs. Patients of both treatment groups were assessed at the time of recruitment (BL), as well as 6 months (FU1, follow-up one) and 12 months (FU2, follow-up two) after BL using comprehensive questionnaires. In order to achieve adequate response rates, patients were compensated with 30 Euros for participating in the study. Using postal reminders, GPs and patients were asked to complete the follow-up assessments.

### Inclusion/exclusion criteria

Patients were included if: (1) they had a body mass index (BMI) ≥ 30 kg/m^2^, (2) they were between 18 and 60 years old and (3) they had sufficient proficiency in the German language. Patients were excluded if they had an acute medical condition (physical or mental) that required prioritized treatment and made study participation impossible according to the attending GP. There were no in- or exclusion criteria for GPs.

### Randomization and blinding

After study inclusion, GPs were sequentially allocated to intervention or control group using a computerized random number generator in an adaptive randomization process (biased coin design) [39]. Patients were blinded to their group allocation. Blinding of GPs towards the treatment groups was not possible since GPs were directly addressed by the intervention. GPs were informed about their group allocation via postal mail with a sealed envelope after the randomization process was completed. Additionally, GPs received recruitment material and consent forms. A research assistant gave the participating GPs instructions about the recruitment material and procedure of the patient recruitment by telephone. Based on the inclusion and exclusion criteria, GPs then identified and recruited eligible patients within their practices. Patients received information about the study and they were asked to participate by their attending GP. Information material and consent forms were identical for patients of both treatment groups. A statistician who was blinded to the group allocation conducted the data analyses.

### Interventions

#### The web-based 5A intervention

The 5A framework offers a simple tool for GPs to improve patients’ weight management counseling in primary care. It provides recommendations for sensitively discussing weight with the patient (“ASK”), assessing health status, comorbidities and causes of weight gain (“ASSESS”), advising on the health benefits of treatment and available treatment options (“ADVISE”), agreeing on weight loss expectations, treatment plan and treatment goals (“AGREE”) and assisting the patient in the continuous process of weight management (“ASSIST”). Rather than focusing on the amount of weight loss, successful weight management is conceptualized as improved overall health and well-being. Based on the 5As guidance for obesity management by the Canadian Obesity Network [28], a short 5A online tutorial was developed. The 5A online tutorial comprises an introduction, five knowledge sections and a short knowledge quiz at the end. While the introduction includes information on learning objectives and basic principles of obesity management, each of the five knowledge sections covers one of the 5A components. For example, the “ADVISE” section contains information on obesity-related treatment options (physical exercise, nutrition, psychotherapy, medication and surgery) and the “AGREE” section covers criteria for defining realistic goals. Table 1 gives an overview of the components of the 5A intervention. The short quiz at the end of the 5A online tutorial consists of seven questions (e.g. “How would you react if a patient told you that he or she doesn’t want to talk about his or her weight?”).

**Table 1:** Overview of the components of the 5A intervention

**5A’s**	**Content**
ASK	• Discuss weight and motivation with the patient
ASSESS	• Assess health status and obesity class, comorbidities and causes of weight gain
ADVISE	• Advise on obesity risks, benefits of weight loss and treatment options
AGREE	• Agree on health outcomes, weight loss expectations and treatment plan
ASSIST	• Assist the patient in the continuous process of weight management and arrange follow-up visits

#### TAU/Control group

Patients whose attending GP was aligned to the CG received treatment as usual (TAU). GPs of the CG were given no constraints regarding TAU. The treatment of obesity was therefore under the sole responsibility of the attending GP.

### Outcomes

#### Primary outcome

Corresponding to the 5A framework, *provider-patient-interaction* regarding the management of obesity was the primary outcome of the INTERACT study. Patients’ perspective on the provider-patient-interaction over the past 6 months was assessed using the German version of the Patient Assessment of Chronic Illness Care (PACIC 5A [40, 41]). The PACIC is a widely used instrument to assess patient-centeredness and quality of care provided to patients with chronic diseases [42–44]. The PACIC 5A is an extended version that furthermore assesses quality of care with regard to the 5A concept. The PACIC 5A consists of 26 items rated on a 5-point Likert scale ranging from 1 (= almost never) to 5 (= almost always). The first 20 items cover the five subscales patient activation, delivery of care, goal setting, problem solving, and follow-up. The remaining 6 items cover the 5A approach. The scoring instruction provided by Rosemann et al. [40] was applied to calculate the PACIC 5A sum score. The PACIC 5A sum score can range from 25 to 125, with higher scores indicating a stronger perceived congruency to the 5A approach.

#### Secondary outcomes

*Weight status* was assessed using the BMI. The BMI was calculated through self-reported height (at BL) and weight (at every point of assessment).

Patients’ *health-related quality of life* was measured using the German version of the EQ-5D-5L [45]. The EQ-5D-5L comprises a visual analog scale (EQ VAS) from 0 (= worst health) to 100 (= best health) and 5 questions which cover perceived impairments in five dimensions of health-related quality of life (mobility, self-care, usual activities, pain/discomfort, anxiety/depression). The five items are rated on a 5-point response scale ranging from 1 to 5 (e.g. 1 = “I have no pain or discomfort”, 5 = “I have extreme pain or discomfort”). The EQ-5D-5L sum score was calculated according to Hinz et al. [46].

Patients’ *willingness to engage in weight management strategies* was measured with an adapted version of the Readiness Ruler [47, 48], a visual analog scale assessing the readiness for a change of lifestyle in order to achieve weight loss on a scale from 0 (= not ready to change) to 10 (= ready to change).

*Depressive symptoms* within the last two weeks were measured with the German version of the PHQ-9 [49, 50]. The PHQ-9 comprises nine items (e.g. “little interest or pleasure in doing things”, “poor appetite or overeating”) to be rated on a 4-point scale ranging from 0 (= not at all present) to 3 (= present nearly every day). The PHQ-9 sum score ranges from 0 (= no depression) to 27 (= severe depression), with higher scores indicating a more severe symptomatology.

*Internalized weight bias* was assessed using the German adaptation of the Weight Bias Internalization Scale (WBIS) [51, 52]. The WBIS comprises 11 Items with a 7-point response scale ranging from 1 (= strongly disagree) to 7 (= strongly agree). Higher scores indicate a stronger internalized weight bias.

*Weight loss intentions and activities of weight management* were assessed using an adapted version of the stages of change algorithm [53, 54]. This algorithm comprises four questions with a yes/no response scale, resulting in four categories of change (precontemplation, contemplation, action, maintenance).

#### Other measures

Apart from sociodemographic characteristics (age, gender, education, and marital status), anxiety symptoms, personality traits and counseling experiences of patients were also assessed. *Anxiety symptoms* were measured using the subscales for ‘panic syndrome’ and ‘other anxiety syndrome’ of the PHQ-D [49]. The subscale for panic syndrome comprises 15 items with a yes/no response scale. The subscale for other anxiety syndrome consists of 7 items scored on a 3-point response scale from 1 (= not at all present) to 3 (= present for more than half the days).

The 10-item Big Five Inventory (BFI-10) [55] was used to assess *personality traits*. The BFI-10 measures five personality dimensions with two items each on a 5-point response scale ranging from 1 (= disagree strongly) to 5 (= agree strongly).

*Counseling experiences* of patients with their attending GP were assessed using four yes/no questions (“Have you seen your GP within the last six months?”, “How often have you seen your GP within the last six months?, “Has your weight been discussed in the consultation and who took the initiative?”, and “Which aspects of weight have been discussed?”).

#### Assessment of GPs

Questionnaires for GPs contained closed questions on referral and counseling behavior of patients with obesity, satisfaction with own knowledge about obesity, attitudes towards obesity as a chronic disease, relevance of different causes of weight gain and attitudes on different aspects of obesity management.

Additionally, *stigma concerning obesity* was assessed using a German adaptation of the short form of the Fat Phobia Scale (FPS) [56, 57]. The FPS comprises 14 pairs of adjectives on a semantic differential (e.g. active = 1 vs. inactive = 5). The FPS sum score was calculated as the mean score of all 14 items. Higher scores indicate higher negative attitudes.

Moreover, GPs from the IG were asked to evaluate the 5A online tutorial concerning the relevance of its knowledge contents and its usability within the primary care setting 12 months’ post-intervention.

### Sample size

According to Rueda-Clausen et al. [31], a 15 point difference prior to post-intervention was regarded as a clinical significant change in the PACIC 5A sum score. With a power of 95%, a standard deviation of 20 points [31] and considering an intraclass correlation coefficient of 5%, we calculated a sample size of n = 86 patients per group to measure an effect at follow-up. Power calculation and sample size have been described in detail in the study protocol [58].

### Statistical analysis

Statistical analyses were conducted using IBM SPSS Statistics (25 SPSS Inc., Chicago, IL, USA) and STATA 13.1 SE software package (StataCorp LP, College Station, TX). Descriptive statistics are presented as number of cases with percentages or means with 95% confidence intervals. Inspection of missing values in outcomes and covariates at BL revealed no systematic patterns in both treatment groups; missing information was thus handled by case wise deletion. Mixed-effects linear regression models were calculated to estimate mean differences between treatment groups in primary and secondary outcomes from BL to FU2. The models included indicators of treatment group (IG vs. CG), time (BL, FU1, and FU2) and the interaction between treatment group and time, and were adjusted for the BL outcome value and confounding factors (i.e., age, gender, education, marital status and neuroticism). Covariates were included based on previous research linking patient characteristics to patient satisfaction with the received health care [59–64]. With regard to personality, neuroticism has been included as a confounding factor as it has been linked to BMI variations over time [65], poorer health outcomes [66], lower psychological well-being, and reduced life satisfaction [67, 68]. Neuroticism may further affect patients’ presentation of symptoms in a way that influences medical care [69]. In order to control for non-random dropout effects, all analyses were intention-to-treat as recommended according to the guidelines of the CONSORT statement [70]. Standard errors were corrected for the clustered trial design using the Huber/White sandwich estimator. Regression analyses results are presented as adjusted mean group differences in outcome scores at follow-up. In addition, we estimated standard effect sizes of treatment on primary and secondary outcomes at follow-up (Cohen’s d). For all statistical analyses, the level of statistical significance was set at *p* < 0.05.

## Results

### Patient flow

**Fig. 1:**
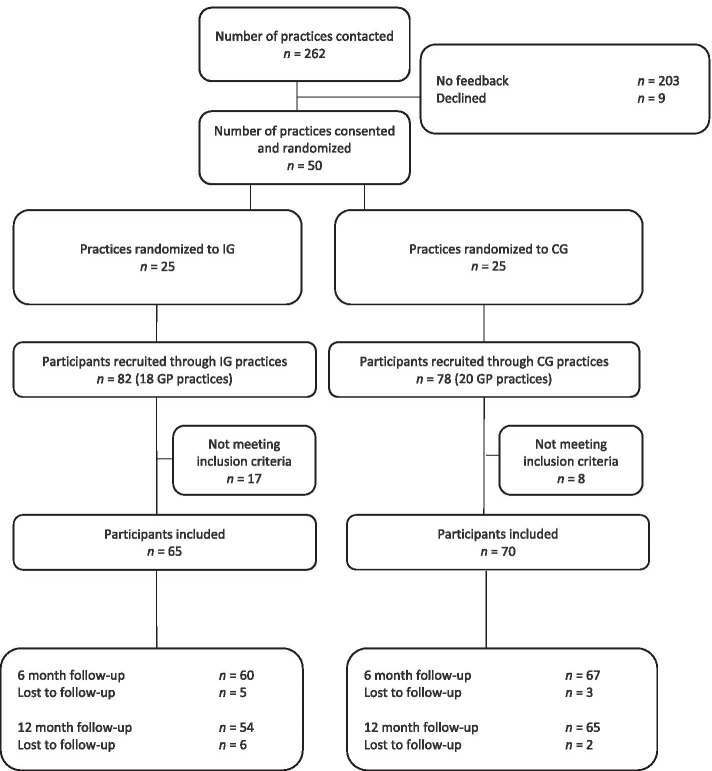
Sample selection flowchart

Out of the 262 general practices contacted, 50 GPs were recruited and randomised to participate in the INTERACT study (IG: *n* = 25, CG: *n* = 25). Of those, 18 GPs of the IG and 20 GPs of the CG recruited participants for the study. In total, 160 patients were recruited through GPs at baseline. After applying inclusion and exclusion criteria, 135 (IG: *n* = 65, CG: *n* = 70) patients were included in the study. Of the 135 patients included in the baseline assessment, 127 patients returned questionnaires at 6-month follow-up (response rate: 94.1%, IG: 92.3%, CG: 95.7%) and 119 returned questionnaires at 12-month follow-up (response rate: 88.1%, IG: 83.1%, CG: 92.9%). Out of the 50 GPs that were recruited, 42 GPs (response rate: 84%, IG: *n* = 19, 76%, CG: *n* = 23, 92%) returned questionnaires at the 12-month follow-up assessment. Figure 1 provides an overview of the sample selection process.

### Baseline characteristics

#### GPs

Recruited GPs were on average 48.6 years old, had an average working experience of 20.6 years and were mostly female (61.2%). GPs of the IG and the CG did not differ with regard to age, working experience, gender distribution and BMI. However, GPs of the IG had higher FPS scores compared to GPs of the CG (IG: M = 3.8, SD = 0.3, CG: M = 3.6, SD = 0.4, *p* = 0.008). Asked, if GPs would wish for more training on obesity counselling, 57.2% agreed with that statement (IG: 52.0%, CG: 62.5%). Regarding the GPs self-evaluation of their expertise on obesity counselling, 77.9% evaluated their expertise in this respect as good or very good (IG: 84.0%, CG: 70.8%), 20.4% as sufficient (IG: 12%, CG: 29.2%), and 2.0% as insufficient (IG: 4.0%). Characteristics of the participating GPs are summarized in Table 2.

**Table 2:** Baseline characteristics of the general practitioner study sample

Variable	Total, *n* = 50	IG, *n* = 25	(95% CI)	CG, *n* = 25	(95% CI)
Gender, n (%)^a^					
Female	30 (61.2)	13 (52.0)		17 (70.8)	
Male	19 (38.8)	12 (48.0)		7 (29.2)	
Age (years), M (SD)^a^ Range: 35 – 79	48.6 (8.8)	48.2 (8.8)	(44.6–51.8)	49.1 (9.0)	(45.3–52.9)
BMI^b^, M (SD) Range: 18.1 – 31.8	23.9 (2.9)	23.6 (3.0)	(22.4–24.9)	24.2 (2.8)	(23.0–25.5)
Work experience (years)^c^, M *(SD),* Range: 3 – 56	20.6 (9.94)	19.5 (9.4)	(15.5–23.5)	21.7 (10.5)	(17.2–26.1)
FPS (sum score), M (SD)^a^	3.7 (0.36)	3.8 (0.32)	(3.7–3.9)	3.6 (0.35)	(3.4–3.7)
Do you wish for more training offers on obesity counseling?^a^					
Agree, n (%)	28 (57.2)	13 (52.0)		15 (62.5)	
Neither agree nor disagree, n (%)	8 (16.3)	5 (20.0)		3 (12.5)	
Disagree, n (%)	13 (26.5)	7 (28.0)		6 (25.0)	
GPs self-evaluation of their expertise on obesity counseling^a^					
Good or very good, n (%)	38 (77.9)	21 (84.0)		17 (70.8)	
Sufficient, n (%)	10 (20.4)	3 (12.0)		7 (29.2)	
Insufficient, n (%)	1 (2.0)	1 (4.0)			

*IG* Intervention group, *CG* Control group, *n* Number of cases, *M* Mean, *SD* Standard deviation, *%* Percent value, *CI* Confidence Interval, *BMI* Body-Mass-Index, *FPS* Fat Phobia Scale, *GP* General practitioner

^a^Missing data for *n* = 1 general practitioner

^b^Missing data for *n* = 3 general practitioners

^c^Missing data for *n* = 2 general practitioners

#### Patients

The included patients were on average 43.3 (SD = 10.7) years old, had an average BMI of 39.0 kg/m^2^ (SD = 6.0) and were more often unmarried (44.0%) than married (36.6%) or divorced or widowed (19.4%). The majority of the sample was female (62.2%) and had a medium level of educational attainment (68.1%). Patients of the IG and the CG were balanced with respect to sociodemographic characteristics, the primary outcome and secondary outcomes at BL. Baseline characteristics of the patient study sample are shown in Table 3. More than four-fifths of patients in both groups (IG and CG) were in the stage of action or maintenance with regard to weight loss intentions and activities of weight management at BL (IG: 91.9%, CG: 86.4%), 6-month follow-up (IG: 84.2%, CG: 83.3%) and 12-month follow-up (IG: 82.3%, CG: 81.3%). Average scores of primary and secondary outcomes at BL and follow-up assessments are presented in Table 4.

**Table 3:** Baseline characteristics of the patient study sample

Variable	Total (*n* = 135)	IG (*n* = 65)	(95% CI)	CG (*n* = 70)	(95% CI)
Age, M *(SD)*	43.3 (10.7)	43.2 (10.1)	(40.7–45.7)	43.3 (11.2)	(40.6–46.0)
Gender, *n (%)*					
Female	84 (62.2)	39 (60.0)		45 (64.3)	
Male	51 (37.8)	26 (40.0)		25 (35.7)	
Level of Education^a^, *n (%)*					
Low	32 (23.7)	13 (20.0)		19 (27.1)	
Medium	92 (68.1)	44 (67.7)		48 (68.6)	
High	11 (8.1)	8 (12.3)		3 (4.3)	
Marital status^b^, n (%)					
Married	49 (36.6)	28 (43.8)		21 (30.0)	
Unmarried	59 (44.0)	25 (39.1)		34 (48.6)	
Divorced/widowed	26 (19.4)	11 (17.1)		15 (21.4)	
PHQ, subscale panic syndrome^c^					
Panic syndrome, n (%)	15 (11.6)	7 (10.8)		8 (12.5)	
PHQ, subscale other anxiety^d^					
Anxiety syndrome, n (%)	6 (4.9)	2 (3.2)		4 (6.8)	
BFI-10^c^, M (SD)					
Extraversion	3.3 (1.1)	3.4 (1.1)	(3.2–3.7)	3.3 (1.0)	(3.1–3.6)
Neuroticism	2.9 (1.0)	2.7 (1.0)	(2.5–3.0)	3.1 (0.9)	(2.9–3.3)
Openness	3.4 (0.9)	3.3 (0.9)	(3.1–3.5)	3.5 (0.9)	(3.2–3.7)
Conscientiousness	3.8 (0.7)	3.8 (0.8)	(3.6–4.0)	3.7 (0.7)	(3.5–3.9)
Agreeableness	3.2 (0.8)	3.3 (0.8)	(3.1–3.5)	3.1 (0.8)	(2.9–3.3)
*Counseling Experience*					
How often have you seen your GP within the last 12 months?^b^ n (%)					
Less than five times	73 (54.5)	34 (53.1)		39 (55.7)	
Five times or more	61 (45.6)	30 (46.9)		31 (44.3)	
Has your weight been discussed?^e^ n (%)					
Yes	106 (79.7)	52 (81.3)		54 (78.3)	
If yes, who took the initiative?^f^ n (%)					
GP	26 (38.2)	14 (42.4)		12 (34.3)	
Patient	22 (32.4)	6 (18.2)		16 (45.7)	
Both (Patient and GP)	20 (29.4)	13 (39.4)		7 (20.0)	
Which aspects of weight have been discussed? n (%)					
Necessity to lose weight	83 (78.3)	41 (78.8)		42 (77.8)	
Health risks	56 (52.8)	29 (55.8)		27 (50.0)	
Secondary diseases	74 (69.8)	37 (71.2)		37 (68.5)	
Diet	61 (57.5)	32 (61.5)		29 (53.7)	
Physical exercise	63 (59.4)	32 (61.5)		31 (57.4)	
Medication	36 (34.0)	15 (28.8)		21 (38.9)	

*IG* Intervention group, *CG* Control group, *n* Number of cases, *M* Mean, *SD* Standard deviation, *%* Percent value, *CI* Confidence Interval

^a^Level of education based on the revised version of the international CASMIN educational classification [71]

^b^Missing data for *n* = 1 participant

^c^Missing data for *n* = 6 participants

^d^Missing data for *n* = 13 participants

^e^Missing data for *n* = 2 participants

^f^Missing data for *n* = 38 participants

**Table 4:** Mean scores of primary and secondary outcomes at baseline, 6-month follow-up and 12-month follow-up

	IG			CG		
	n	M	(95% CI)	n	M	(95% CI)
**Primary Outcome**						
PACIC 5A sum score						
Baseline	56	58.0	(52.3–63.7)	61	56.3	(50.7–62.0)
6 months	53	60.5	(53.8–67.2)	59	54.9	(49.2–60.7)
12 months	50	59.0	(52.7–65.2)	61	52.5	(46.7–58.4)
**Secondary Outcomes**						
BMI						
Baseline	63	39.0	(37.5–40.5)	69	38.9	(37.5–40.3)
6 months	58	37.9	(36.3–39.5)	66	38.8	(37.2–40.4)
12 months	53	38.0	(36.4–39.7)	64	38.4	(36.7–40.0)
EQ-5D-5L						
Baseline	64	84.3	(80.6–88.0)	69	79.5	(75.4–83.6)
6 months	59	83.6	(79.6–87.7)	65	80.8	(76.3–85.3)
12 months	51	85.9	(81.9–89.9)	64	81.5	(77.0–85.9)
Readiness Ruler						
Baseline	65	7.68	(7.20–8.16)	69	7.82	(7.32–8.31)
6 months	59	7.20	(6.62–7.78)	67	7.78	(7.23–8.33)
12 months	52	7.10	(6.49–7.69)	64	7.23	(6.56–7.90)
WBIS						
Baseline	65	3.78	(3.45–4.10)	69	3.84	(3.53–4.14)
6 months	56	3.29	(2.96–3.61)	63	3.88	(3.57–4.20)
12 months	53	3.55	(3.16–3.94)	61	3.66	(3.35–3.98)
PHQ-9						
Baseline	63	6.52	(5.36–7.69)	68	7.41	(6.13–8.69)
6 months	57	5.51	(4.40–6.62)	65	6.49	(5.31–7.68)
12 months	50	5.20	(4.03–6.37)	63	6.86	(5.52–8.19)
	n	%		n	%	
**Stages of Change**						
Precontemplation						
Baseline	3	4.8		1	1.5	
6 months	6	10.5		5	8.3	
12 months	6	11.8		5	8.5	
Contemplation						
Baseline	2	3.2		8	12.1	
6 months	3	5.3		5	8.3	
12 months	3	5.9		6	10.2	
Action						
Baseline	40	64.5		39	59.1	
6 months	39	68.4		35	58.3	
12 months	37	72.5		37	62.7	
Maintenance						
Baseline	17	27.4		18	27.3	
6 months	9	15.8		15	25.0	
12 months	5	9.8		11	18.6	

*IG* Intervention group, *CG* Control group, *n* Number of valid cases, *M* Mean, *CI* Confidence interval, *%* Percent value

### Intention-to-treat analysis

#### Primary outcome

Results for the primary outcome showed no significant group differences at 6-month and 12-month follow-up for the PACIC 5A sum score. Results from the mixed-effects linear regression are shown in Table 5.

**Table 5:** Results from the mixed-effects linear regression models for primary and secondary outcomes

Mixed model^a^						
Outcome	n	Diff	(95% CI)	*p*	d	(95% CI)
**Primary Outcomes**						
PACIC 5A sum score						
Baseline	113	0.03	(-2.37–2.44)	.976		
FU1 – 6 months after BL	100	2.53	(-4.97–10.03)	.509	0.12	(-0.27–0.52)
FU2 – 12 months after BL	101	2.81	(-2.45–8.08)	.295	0.20	(-0.18–0.60)
**Secondary Outcomes**						
BMI						
Baseline	125	-0.15	(-0.52–0.23)	.450		
FU1 – 6 months after BL	115	-0.62	(-1.43–0.19)	.134	-0.29	(-0.65–0.77)
FU2 – 12 months after BL	109	-0.22	(-1.10–0.65)	.617	-0.95	(-0.47–0.28)
EQ-5D-5L total score						
Baseline	126	0.21	(-0.92–1.33)	.717		
FU1 – 6 months after BL	115	-0.34	(-3.90–3.22)	.851	-0.03	(-0.40–0.33)
FU2 – 12 months after BL	109	1.28	(-2.25–4.82)	.476	0.13	(-0.24–0.51)
Readiness Ruler						
Baseline	127	0.07	(-0.16–0.29)	.559		
FU1 – 6 months after BL	118	-0.61	(-1.19—-0.03)	< .05	-0.39	(-0.75—-0.02)
FU2 – 12 months after BL	110	-0.26	(-1.07–0.55)	.529	-0.11	(-0.49–0.26)
WBIS						
Baseline	128	0.02	(-0.10–0.15)	.699		
FU1 – 6 months after BL	113	-0.49	(-0.82—-0.15)	< .01	-0.53	(-0.90—-0.15)
FU2 – 12 months after BL	108	-0.16	(-0.47–0.15)	.308	-0.19	(-0.57–0.18)
PHQ-9 total score						
Baseline	125	-0.12	(-0.56–0.32)	.586		
FU1 – 6 months after BL	113	-0.22	(-1.15–0.71)	.646	-0.08	(-0.45–0.28)
FU2 – 12 months after BL	106	-0.69	(-2.05–0.65)	.310	-0.19	(-0.58–0.18)

*Diff* Adjusted mean outcome differences, i.e., intervention group minus control group, *CI* Confidence interval, *FU1* Follow-up 1, *FU2* Follow-up 2, *d* Effect size: standardized mean difference (Cohen’s d) at follow-up

^a^Mixed-effects linear regression of outcome on treatment group (intervention group vs. control group), time (baseline, FU1, FU2) and interaction between treatment group and time adjusted for baseline score, age, gender, education, marital status and neuroticism

#### Secondary outcomes

Results for secondary outcomes showed no significant group differences for any of the outcomes with the exception of the Readiness Ruler and the WBIS at 6-month follow-up (see Table 5). Adjusted for the scores at BL and further covariates, participants of the IG scored on average 0.6 points (*p* = 0.037) lower on the Readiness Ruler and 0.5 points (*p* = 0.004) lower on the WBIS than participants of the CG. The estimated size of the effect was small for the Readiness Ruler (d = 0.4) and medium for the WBIS (d = 0.5). However, adjusted mean differences for both outcomes did not significantly differ at 12-month follow-up.

### Uptake, adherence and acceptability of the 5A online tutorial

#### General attitudes towards e-health interventions

Considering the application of web-based tutorials in general, 47.4% (*n* = 9) of the GPs allocated to the IG disagreed with the statement that new media like the internet should be more strongly involved in obesity therapy, whereas 42.1% (*n* = 8) of the GPs agreed and 10.5% (*n* = 2) neither disagreed nor agreed. Concerning personal knowledge about web-based applications, 47.4% (*n* = 9) of the GPs stated that they only had little knowledge about web-based tutorials, while 47.4% (*n* = 9) disagreed with that statement and 5.2% (*n* = 1) neither agreed nor disagreed. Only 7 out of 19 GPs (36.8%) agreed with the statement that they would like to get to know more about web-based tutorials for obesity treatment, while 9 (47.4%) disagreed and 3 (15.8%) neither agreed nor disagreed.

#### Uptake and adherence

Of all GPs with access to the 5A online tutorial (*n* = 25), 76% (*n* = 19) completed the 5A online tutorial entirely, requiring an average time of 35 min. Another 16% (*n* = 4) terminated the 5A online tutorial prematurely after 19 min on average, and 8% (*n* = 2) of the GPs did not access the 5A online tutorial at all.

#### Acceptance of the 5A online tutorial

With respect to the 5A online tutorial (relevance of its knowledge contents and usability within the primary care setting), 63.2% (*n* = 12) of the GPs in the IG agreed with the statement that the tutorial comprised exactly the issues which are relevant for obesity treatment and counseling, while 26.3% (*n* = 5) disagreed and 5.3% (*n* = 1) neither agreed nor disagreed. Regarding the statement that the 5A online tutorial is a useful addition for an optimized treatment of obesity, 57.9% (*n* = 11) agreed, 26.3% (*n* = 5) disagreed and 5.3% (*n* = 1) neither agreed nor disagreed. Similarly, 63.2% (*n* = 12) agreed that the 5A online tutorial can help treatment providers to start a conversation about weight with patients with obesity, while 26.3% (*n* = 5) disagreed and 5.3% (*n* = 1) neither agreed nor disagreed. Table 6 shows the utilization assessment results for GPs of the IG.

**Table 6:** Evaluation of eLearning and the 5A tutorial by general practitioners (IG only, *n* = 19)

Variable	*n (%)*						
	Strongly disagree	Disagree	Rather disagree	Neither disagree nor agree	Rather agree	Agree	Strongly agree
*Application of web-based tutorials*							
I think new media like e.g. the internet should be more strongly involved in obesity therapy	1 (5.3)	4 (21.1)	4 (21.1)	2 (10.5)	3 (15.8)	5 (26.3)	-
I have little knowledge about web-based tutorials so far	2 (10.5)	3 (15.8)	4 (21.1)	1 (5.3)	4 (21.1)	2 (10.5)	3 (15.8)
I would like to get to know more about web-based tutorials for obesity treatment	3 (15.8)	4 (21.1)	2 (10.5)	3 (15.8)	3 (15.8)	2 (10.5)	2 (10.5)
*Evaluation of the 5A online tutorial*							
The 5A online tutorial comprises exactly the issues which are relevant for the treatment and counselling of patients with obesity^a^	-	4 (21.1)	1 (5.3)	1 (5.3)	6 (31.6)	3 (15.8)	3 (15.8)
The 5A online tutorial is a useful addition for an optimized treatment of obesity^b^	-	3 (15.8)	2 (10.5)	1 (5.3)	3 (15.8)	3 (15.8)	5 (26.3)
The 5A online tutorial can help providers to start a conversation with their patients about weight^c^	1 (5.3)	2 (10.5)	2 (10.5)	1 (5.3)	5 (26.3)	3 (15.8)	4 (21.1)

*IG* Intervention group, *n* Number of cases

^a^Missing data for *n* = 1 participant

^b^Missing data for *n* = 2 participants

^c^Missing data for *n* = 1 participant

## Discussion

The aim of the INTERACT study was to evaluate an online tutorial for GPs based on the 5A framework of obesity management with respect to the quality of the provider-patient interaction*.* The INTERACT study provides the first long-term results for a minimal 5A-based intervention in the context of the German primary care setting. The results showed no treatment effect of the 5A tutorial regarding the quality of weight counseling based on the patients’ perspective at 6-month and 12-month follow-up.

### Effectiveness of 5A-based counseling in primary care

Contrary to our results, several studies have found improvements in counseling performances [31, 32] or a reduction in patients’ weight [35] related to the 5As counseling approach. However, comparability of those findings with the findings of our study is limited due to differences concerning study design and intervention implementation. Previous studies mostly used comprehensive face-to-face teaching methods or implemented a combination of face-to-face training and computer-assistance [32, 34, 35]. In the present study, however, a minimal e-learning intervention was implemented that took less than 40 min to complete for participating GPs. Thus, the intervention had the benefit of being easily integrated in already busy work schedules of the GPs. However, the intervention may have been too low in training intensity or may have needed intermediate refresher trainings. Similar to our study, Rueda-Clausen et al. [31] implemented a short online intervention on 5A counseling for GPs and found improvements in the counseling performance regarding weight management from the patients’ perspective. Yet, the study reported only short-term results from a non-controlled quasi-experimental design [31]. In short, although the 5As are a simple and minimal intervention to improve GP counseling, performances may have primarily short-term effects on the quality of weight management and the provider-patient interaction while failing to maintain positive counseling effects in the long-term without continuing training-sessions. Furthermore, methodological problems may be further reasons for our null results, as the sample size may have been slightly underpowered due to difficulties with patient recruitment through GPs. We estimated that we would need a sample size of *n* = 66 patients per group at 12-months follow-up [58]. While we nearly achieved this number in the control group (*n* = 65), we missed that target in the intervention group (*n* = 54). Further, due to missing values in the primary outcome the sample size may have been too low to detect a real effect.

With regard to quality of life, we did not find any effects at 6-months or 12-months follow-up. Given the null results on the primary outcome of perceived quality of care as well as unchanged weight states of patients, this is not a surprising result. However, we used a general measure of health-related quality of life (EQ5D), which may have not been sensitive enough to detect changes in our sample of patients with obesity. While the EQ5D has been frequently used to assess quality of life in individuals with overweight or obesity [72–74], it may be rather poor in subsamples of people with pronounced obesity [75].

### Characteristics of patients

With regard to patients’ characteristics, both groups in our study were well balanced. Still, as was shown by the results of the stages of change algorithm and the Readiness Ruler, patients’ willingness to engage in weight management strategies at baseline was high in both groups with mean values above 7.8 on a scale of 0 to 10. According to the Transtheoretical Model (TTM) [76–78] the stages of change describe five successive stages of an individual changing from an unhealthy to a more healthy behavior: Precontemplation, Contemplation, Preparation, Action, and Maintenance. In correspondence to the TTM, tailoring weight counseling to the individual patient initially requires the assessment of readiness, motivation and confidence in behavior change. Following, appropriate communication strategies should be selected that correspond to the individual’s stage of change [79]. General recommendations on how to address each of the five stages have been established previously [80, 81]. The 5A framework may be specifically suitable to structure that process of communication because it encourages physicians to assess the motivation of the patient and give feedback on the health status first, addressing weight management subsequently based on the patient’s readiness and health status. However, the high readiness to engage in weight management that we found in both groups at baseline may have further hindered the detection of significant changes in obesity outcomes due to a ceiling effect of weight loss motivation. Brief weight counseling interventions in primary care may be less effective in patients who are already highly motivated. Future studies should consider screening for weight loss motivation before study inclusion to achieve higher variability in this respect.

### User acceptance of the intervention

Research has previously demonstrated that the effectiveness of internet-based learning is comparable to that of traditional face-to-face learning modalities in health professions education [82]. User acceptance for e-learning modalities is a vital precondition for such web-based interventions to be effective and integrated in clinical practice. With regard to the context of obesity treatment, we found that GPs’ assessments of e-learning applications were fairly balanced between approval and disapproval. About half of the GPs allocated to the IG indicated that they had only little knowledge about web-based tutorials in general and only about a third of the GPs indicated that they would like to get to know more about web-based tutorials for obesity treatment. These results are in line with a relatively low usage of e-learning programs for educational purposes [83] and a preference for traditional settings (e.g. face-to-face education) for continuing medical education by German physicians [84]. Still, in the present study the 5A online tutorial was rated positively by the majority of participating GPs with almost two-thirds stating that the tutorial covered relevant issues for obesity treatment and more than half of the GPs perceiving the 5A tutorial as a useful addition for optimized obesity treatment. Then again, about one-third of the GPs in the IG who returned questionnaires at 12-month follow-up did not perceive the 5A intervention as a relevant or helpful tool for obesity treatment. Therefore, the non-significant intervention effects may still be reflective of a limited user acceptance for the online intervention. However, the number of GPs participating in the intervention and returning questionnaires was small and the results may not be representative of GPs in general. Future studies need to shed more light on reasons behind a reduced user acceptance for minimal e-learning interventions in health professions education.

### Characteristics of GPs

Barriers to quality counseling in primary care have been reported on the level of GPs and patients characteristics. While female gender of the GPs has been associated with higher quality of obesity counseling in previous research [33], gender differences may not explain the results in our study. Both study groups, IG and CG, have been fairly balanced with regard to age, gender and working experience of the GPs. However, there was a significant difference between GPs of both groups with respect to stigma. GPs of the IG appeared to have had stronger negative attitudes towards obesity than GPs of the CG. While the reasons for this difference between both groups remain unclear, previous research has shown that the experience of stigmatization is associated with impaired psychological functioning and eating behavior [85]. Furthermore, in the context of medical care stigma has been shown to influence treatment recommendations by GPs [86]. In the present study, differences in stigma between both groups may have influenced the quality of obesity counseling and may therefore be a further contributing factor to the non-significant results.

### Strengths and limitations

Strengths of the INTERACT study include the cRCT design, which forms a framework for evaluating causal effects and a high extent of external validity through its implementation within the day-to-day management of primary care. Data were collected using reliable and valid instruments. Furthermore, patients from the IG and the CG did not differ with respect to the primary outcome and secondary outcomes, nor did they differ with respect to relevant covariates (e.g. age, gender, education) at baseline. In sum, there was a satisfactory balance between intervention and control group, minimizing the chances for a possible sampling bias. Finally, the implementation of the 5A online tutorial has been overall acknowledged by most of the participating GPs, with 76% completing the tutorial.

However, there are also a number of limitations to be considered. First, no information was available on the motives of GPs not completing the tutorial, while this information could be useful for improving user acceptance in the future. Second, due to the study design, blinding of GPs towards the treatment group was not possible. Additionally, due to recruitment difficulties, the patient sample was smaller than originally intended and some patients had to be excluded after previous inclusion through their attending GP because they did not fulfill the inclusion criteria. Hence, the study sample may have been underpowered. However, response rates were high in patients of both groups with less than 17% dropout at the second follow-up assessment. Another limitation is the use of self-reported height and weight to calculate the BMI. Specifically, self-reported weight tends to be under-reported in individuals with overweight or obesity [87]. However, recent studies suggested self-reported height and weight to be overall valid measures to calculate the BMI because misclassifications are reasonably small [88, 89]. The results may further lack generalizability as patients aged 61 years or older were excluded from study participation. Older adults were excluded from participation for two reasons. First, recommendations for optimal BMI and the treatment of obesity in later life may distinguish from young and middle-aged adults as older adults often present with more comorbidities and chronic diseases [90–92]. Second, specifically old age of the patient has been reported to moderate the relationship between the physician–patient interaction and the patient satisfaction [93–95]. Furthermore, GPs of both groups differed with regard to their extent of stigma towards patients with obesity. GPs of the IG showed higher stigma towards obesity, therefore potentially undermining the possible effectiveness of the intervention. Finally, the majority of GPs with access to the intervention rated the 5A online tutorial as a useful and relevant tool in the treatment of obesity. However, attitudes towards online tutorials in general as a supplement for obesity management were fairly balanced between approval and disapproval indicating the possibility of limited user acceptance for the online intervention.

## Conclusions

To our knowledge, the INTERACT study provides the first long-term data for a 5A-based minimal e-learning intervention for obesity counseling in the German primary care setting. The results suggest that a low-threshold minimal e-learning intervention for GPs does not improve weight management in the long term without further reminder or professional support. Given the negative attitudes of many GPs towards patients with obesity, future studies should also consider the role of these attitudes in the context of web-based interventions for obesity counseling. Future research should further expand the present results by enhancing the user acceptance of online tutorials among GPs. To improve weight management in primary care, comprehensive strategies are needed. Brief online tutorials may serve as one tool in a comprehensive strategy. If online tutorials are considered, improving user acceptance among GPs may also include booster sessions between follow-up assessments in order to intensify the intervention. Since more than half of the GPs wish for more training offers on obesity counseling, there is a clear need for further research in this area.

## Data Availability

Due to ethical restrictions and patient confidentiality issues, detailed patient data cannot be made available publicly. Electronic research data will be made accessible to interested researchers upon reasonable request after signing a non-disclosure agreement.

## Abbreviations

WHO: World Health Organization
GP: General practitioner
cRCT: Cluster-randomized controlled trial
IG: Intervention condition
CG: Waiting list condition
ISAP: Institute of Social Medicine, Occupational Health and Public Health of the University of Leipzig
CME: Continuing medical education
BL: Baseline
FU: Follow-up
BMI: Body mass index
TAU: Treatment as usual
PACIC 5A: Patient Assessment of Chronic Illness Care
EQ-5D-5L: 5-Level EQ-5D version
EQ VAS: Visual analogue scale of the EQ-5D
PHQ: Patient Health Questionnaire
WBIS: Weight Bias Internalization Scale
BFI: Big Five Inventory
FPS: Fat Phobia Scale
TV-L: Collective labour agreement for Federal States
SD: Standard deviation
